# History and Pathology of Vaccination

**Published:** 1890-06

**Authors:** 


*History and Pathology of Vaccination.* By EDGAR M. CROOKSHANK, M.B. Two volumes. Pp. 1076. London: H. K. LEWIS. 1889.

As a specimen of book-making, in the fullest sense of the word, the work before us is a highly favourable example. We can scarcely venture to adjudicate the relative merits of publisher and author in their joint production, so well have both fulfilled their purposes. To the former we are indebted for handsome bindings, exquisite paper, clear and handsome type, and a general get-up which makes the work a sort of *édition de luxe.* To the latter we are indebted for a collation and reprinting of most of the early and historic essays on vaccination and on immediately allied subjects, which must have demanded much industry and a keen interest in the subject. Volume I., entitled "A Critical Inquiry," would more correctly be described as a running commentary on the history of vaccination, with abundant extracts from the writings of others. Volume II. is composed entirely of selected essays, including Jenner's original *Inquiry*, with those of Pearson, Woodville, Loy, Rogers, Birch, Estlin, Ceely and others.

The author is an opponent of vaccination. Now, we are not going to review the work from the point of view of its aims—others more competent have done, and probably will do, this—but we must say something of its methods.

The first peculiarity which strikes us is, that the author shows some want of experience in dealing with scientific memoirs as put forth from eighty to a hundred years ago. In these days of cheap paper and printing, verbosity is neither expensive nor wrong. A writer may now maunder on for pages over the shape of a micrococcus or the peculiarities of a pulse-tracing, and may imagine that, far from being a mere recording machine, he is an experimental philosopher. Such a man would gleefully hack to pieces Newton's *Principia*, and place on the same level the invention of the differential calculus and a discovery of a new aperient. We are very far from saying that the author would be guilty of either sin; but we cannot fail to observe that he expects in Jenner's papers fuller details than scientific men then put into their writings. Jenner's statements are condensed, his sentences are few, the language simple, but they go straight to the point, and at the same time bear the imprint of philosophic observation and study. So highly do we estimate the character of Jenner's work, that we believe we do not unduly depreciate the author's when we say that there is more scientific originality and philosophic grasp in the first four paragraphs of Jenner's original *Inquiry* than in the whole of the author's contribution to the question.

Suppose we admit that the whole theory of vaccination is founded on error, what about the practice and the facts that have accumulated round it? Even if the theory is nonsense, the facts may be unassailable. Many sound practices have originated on false hypotheses, and this may be one of them.

In this work the facts of vaccination are not disputed: they are simply ignored. Now, it is just the practice that we want discussed. By all means let us go back and work out a truer hypothesis if we can; but, in all seriousness, what is the use, in this practical age, of seeking to demolish the almost unanimous evidence of thousands of competent men by a critical study of the scientific writings of early philosophers?

If the author, before stating his belief in the fallaciousness of vaccination, were to spend six months in a smallpox hospital amidst vaccinated nurses and residents, and vaccinated and unvaccinated patients, and were then to tell us that vaccination was useless, we might put some value on his statement. This is the *crux* of the whole question, and it has not even been approached. In the meantime, while deploring the results which the work may produce in the hands of the thoughtless and the ignorant, we cannot but be grateful to the author for bringing together in so convenient and pleasing a form the most important memoirs dealing with the origin of vaccination.

